# Effect of *Moringa oleifera* leaf extract gel on pulp repair following pulpotomy: an in-vivo study

**DOI:** 10.1038/s41598-026-41452-z

**Published:** 2026-03-25

**Authors:** Sabah M. Sobhy, Menna Allah Salem Ali, Aytallah Salem, Hala Ahmed Abd El Moneim Khoriba, Ashraf M. Abu-Seida, Heba Abdelfatah

**Affiliations:** 1 Endodontic Department, Faculty of Dental Medicine for Girls, Al-Azhar University, Cairo, Egypt; 2 Department of Conservative Dentistry, Faculty of Dental Medicine for Girls, Al-Azhar University, Cairo, Egypt; 3 Department of Pedodontics and Oral Health, Faculty of Dental Medicine for Girls, Al-Azhar University, Cairo, Egypt; 4 Department Conservative Dentistry, Faculty of Oral and Dental Medicine, Badr University in Cairo (BUC), Cairo, Egypt; 5 Department of Surgery, Anesthesiology and Radiology, Faculty of Veterinary Medicine, Cairo University, Giza, 12211 Egypt; 6 Oral and Dental Biology, Faculty of Dental Medicine for Girls, Al-Azhar University, Cairo, Egypt; 7 Oral and Dental Biology, Faculty of Oral and Dental Medicine, Nile Valley University, Faiyum, Egypt

**Keywords:** Moringa gel, MTA, Pulpitis, Pulpotomy, Pulp repair, Rabbits, Incisors, Diseases, Health care, Medical research

## Abstract

*Moringa oleifera* (MO) is an herbal agent that has antimicrobial, antioxidant, and anti-inflammatory properties. It may promote reparative dentin formation and resolute pulpal inflammation. However, limited researches have shown their influence on pulpal inflammation. Thus, this study investigated the histological response of the pulp after using of MO in pulpotomy of rabbit’s incisors. For this investigation, twelve adult male New Zealand white rabbits with a total of 48 teeth-two upper and two lower central incisors-were chosen. On the cervical third of the incisors’ labial surface, class V cavities were created. 5 µL of *Escherichia coli* (*E. coli*) lipopolysaccharide O111:B4 at a dosage of 10 mg/mL was used to cause pulpitis following pulp exposure. Glass ionomer was then used to seal the cavities. The pulp was reaccessed and a pulpotomy was carried out following a 24-h period of pulpitis induction. Based on the pulpotomy agent employed, the cavities were randomized into three groups (16 teeth each): group I received white mineral trioxide aggregate (MTA) as a positive control, group II received nothing (negative control), and group III received 15% MO leaf extract gel. To assess pulpal inflammation and repair processes, a histological study was conducted 2 weeks following pulpotomy. A statistical analysis of the data was conducted. Comparing 15% MO leaf extract gel to control groups, there was a statistically significant decrease in the inflammatory cells infiltration and the disorganization of pulp tissue (*P* = 0.000). The 15% MO leaf extract gel and MTA did not significantly differ in terms of reparative dentin development. In contrast to the negative control, both groups displayed a statistically significant difference (*P* = 0.000). Using 15% MO leaf extract gel as a pulpotomy agent results in almost similar new hard tissue formation and less inflammation and pulpal tissue disorganization when compared to white MTA.

## Introduction

Dental caries, despite being preventable, remains a chronic infectious condition that causes health problems around the world. Caries account for 34.1% of the global disease load and remain the largest cause of tooth loss^1^. The World Health Organization (WHO) reports that 60–90% of school-aged children and nearly all adults are affected by dental caries^2^. Managing deep caries lesion in young adults with incomplete root development poses challenges for dentists. This is especially true for first permanent molars which are vulnerable to early caries attack following eruption, play an important role in healthy development of the dentition^3^. Indirect pulp capping, selective removal of carious tissue, or stepwise excavation in deep caries treatments can all reduce the likelihood of pulpal exposure^4^.

The European Society of Endodontology (ESE) and American Association of Endodontists (AAE) state that in cases where vital teeth have extremely deep caries lesion, penetrating the full dentin thickness, renders pulp exposure unavoidable and vital pulp therapy (VPT) is advised. In immature teeth, the goal of VPT is to maintain the vital function of dental pulp allowing for both dentinogenesis and complete root development through apexogenesis. The pulp therapy should stimulate pulp tissue regeneration and protect against future bacterial insult. As a result, selecting a suitable biomaterial for VPT is highly critical and demanding^5,6^. Treatment options for vital pulps includes indirect pulp capping, direct pulp capping, partial pulpotomy, and complete pulpotomy^7^.

There are numerous benefits for using pulpotomy as a definitive treatment not only because it preserves the pulp vitality, but also, it allows the dentin-pulp complex to keep its defensive function by producing a mineralized barrier that protect against further insults^8^. Compared to root canal treatment, pulpotomy is a simpler, faster, and more cost-effective procedure. It does not require complete radicular preparation, therapy minimizing structural weakening of the tooth^9^. Both ESE and AAE have stated that VPT can be utilized in mature teeth with symptoms of pulpitis. Prolonged bleeding following pulp exposure may reflect severe pulpal inflammation and indicate full coronal pulpotomy with applying the wound dressing material at the canal orifices level after achieving hemostasis^5,7^.

Ideal VPT materials should be biocompatible, seal well, be easy to handle and promote dentin bridge formation. Various materials have been proposed for use in pulpotomy, including calcium hydroxide, white and grey MTA, dentine bonding agents, bioactive glass. Several studies have been conducted and compared these materials as pulp capping and pulpotomy agents in humans and animals’ models^10–13^. Calcium hydroxide, a commonly used alkaline material with a pH of around 12.5, which has a bactericidal action and allows the formation of hard tissue. However, there are concerns regarding its potential toxicity due to its caustic action, lack of adhesion to dentin, susceptibility to degradation over time, and the presence of tunnel like defects in the reparative dentin bridge^14^.

Mineral Trioxide Aggregate (MTA), the first generation of hydraulic calcium silicate cements was introduced into dentistry in 1993. It has shown excellent biocompatibility, remarkable sealing qualities and antibacterial activity attributed to its high alkaline pH and the release of calcium hydroxide which support apatite production and dentinogenesis, However, MTA has difficult handling characteristics and prolonged setting time. Furthermore, several investigations reported the potential of grey MTA to discolor teeth^15^.

The popularity of using natural agents in dentistry is increasing nowadays. In dentistry, herbal medicines have been accepted by almost all around the world. MO is a medicinal herb known for its therapeutic properties^16^. MO has been shown to have antimicrobial, antioxidant, and anti-inflammatory effects indicating its usefulness in managing and preventing various oral health issues such as periodontal disease, dental caries, oral ulcers, and mucosal lesions. MO may also help in lowering the levels of pro-inflammatory mediators and inhibiting prostaglandin production^17,18^. Its extract proved to have a high remineralizing potential on demineralized enamel of primary teeth^19^. It is biocompatible and reduces intra-radicular dentin erosion and might be promptly incorporated into endodontic irrigants^20,21^.

Although Moringa oleifera has been studied for its antimicrobial, anti-inflammatory, and regenerative properties in oral tissues^20,21^, its application as a pulpotomy agent in situations involving inflamed pulp has not yet been assessed in vivo. The majority of earlier research on animal pulp was carried out in healthy pulp conditions, which is not representative of clinical situations where pulpotomy is carried out in pulps that are inflamed because of caries. Therefore, inducing pulpitis prior to pulpotomy represents a novel and clinically relevant aspect of this study.

We hypothesized that MO extract may have the potential to reverse or arrest pulpal inflammation and preserve the pulpal tissue. Therefore, the present study histologically assessed pulp repair after using MO in pulpotomy of rabbit’s incisors compared to MTA. The null hypothesis stated that there would be no difference in pulpotomy outcome using 15% MO gel compared to the control groups.

## Methods

### Ethical approval

The present in vivo study was conducted as a randomized within-animal experimental study in accordance with the Animal Research: Reporting of In Vivo Experiments (ARRIVE) guidelines^22^. Ethical approval was obtained from the Research Ethics Committee (REC) of the Faculty of Dental Medicine of the Faculty of Dental Medicine for girls, Al-Azhar University, Cairo, Egypt under the approval code: REC-PD-25-13. All methods were carried out in accordance with the relevant guidelines and regulations.

### Animal model

Twelve adult male New Zealand white rabbits with a total of 48 teeth-two upper and two lower central incisors-were selected. The used rabbits ranged in weight from 3.5 to 4 kg and in age from 10 to 12 months. The rabbits were accommodated in separate galvanized cages (90 cm × 60 cm × 40 cm) and maintained at a room temperature range of 20–25 °C. They were kept under standard conditions and provided with suitable diet as well as fresh water.

### Sample size calculation

Sample size was calculated using G*Power version 3.1.9.7 based on a prior study^23^. A power analysis was used to ensure sufficient power for a two-sided statistical test to reject the null hypothesis of no difference between groups. Using an alpha level of 0.05 and a beta of 0.2, power = 80% and an effect size (f) of (0.475) determined by previous study results. The estimated sample size (n) was (48), with 16 in each group to identify for different hard tissue formation and anti-inflammatory effect of the tested material.

### Sample classification

According to the pulpotomy agent employed, a total of 48 teeth were randomly assigned to three groups (16 teeth each). Group I received white MTA (ProRoot; Dentsply maillefer, Charlotte, North Carolina, USA) as a positive control, Group II received nothing (negative control), and Group III received 15% MO leaf extract gel.

### Preparation of 15% MO leaf extract gel

MO leaf extract gel was prepared by sterilizing, drying, and grinding it into powder. The powder was extracted with a Soxhlet extractor with 95% ethanol as solvent. The extract was put into a flash evaporator. To make 15% MO leaf extract gel, 7.5 gm of MO leaf extract was dissolved in 5 ml of distilled water. Then, 50 gm of sodium carboxymethylcellulose gel base was added gradually to the mixture to achieve homogeneity^24^.

### Surgical procedures

Each rabbit was anaesthetized by intramuscular injection of Xylazine HCl (Xylaject® 2%, ADWIA Co., Egypt) at a dose of 5 mg/kg B.W and Ketamine HCl (Ketamine® 5%, Sigma-Tec Co., Egypt) at a dose of 35 mg/kg B.W. All procedures were conducted under aseptic condition. The teeth were disinfected with 0.2% chlorhexidine solution and rubber dam was used for isolation. Class V cavities were prepared on the cervical third of the labial surfaces of the upper and lower central incisors using # 1 sterile carbide round bur (Mani, Inc, Japan) attached to a low-speed hand piece connected to a micromotor (Strong, SB-LS4C022A, Korea). Pulp horn was mechanically exposed in the center of pulpal floor under copious water irrigation.

Pulpitis was induced after pulp exposure by 5 µL of *E. coli* lipopolysaccharide (LPS) O111:B4 (Sigma Chemical Co., St. Louis, MO, USA) at a concentration of 10 mg/mL. The cavities were then sealed with glass ionomer. After 24 h of pulpitis induction; the pulp was re-accessed, coronal pulp tissue was extirpated and hemostasis was achieved with sterile moistened cotton pellet. After pulpotomy, the cavities were randomly assigned into three groups using simple randomization procedure using a random number generator (https://www.random.org/) as follow:

*Group I* the pulp stump was covered with white MTA (ProRoot; Dentsply maillefer) that was mixed following the manufacturer’s guidelines (1/3 water powder ratio), delivered to the pulp stump using an amalgam carrier and gently adapted with a moistened cotton pellet. A moistened cotton pellet was then placed to maintain the necessary humidity for the proper setting of MTA.

*Group II* no pulpotomy agent and was used.

*Group III* the pulp stump was covered with 15% MO leaf extract gel that was carried to the pulp stump by small ball burnisher. All the cavities were sealed with a sterile cotton pellet and glass ionomer cement (Medifil, ProMedica, Germany) for 2 weeks after the treatment. Then, the rabbits were euthanized with a lethal dose of 100 mg/kg Pentobarbital sodium intravenous injection^23^.

### Histological evaluation

After euthanization, the teeth and the surrounding alveolar bone were carefully dissected and fixed for seven days using 10% buffered neutral formaldehyde (Sigma, St. Louis, USA) before being rinsed in running water and decalcified with an increasing concentration of ethylenediaminetetraacetic acid (EDTA) solution at 4 °C for 2.5 months. The specimens were then washed under running water for 24 h and dehydrated through ascending ethyl alcohol concentrations (ranging from 50 to 100%). Specimens were transferred to xylol to be cleared of alcohol, embedded in paraffin blocks and cut into labiopalatal sections (6 µm thickness) and at 70 μm intervals using a microtome (Leitz 1512, Germany)^25^.

**Fig. 1:**
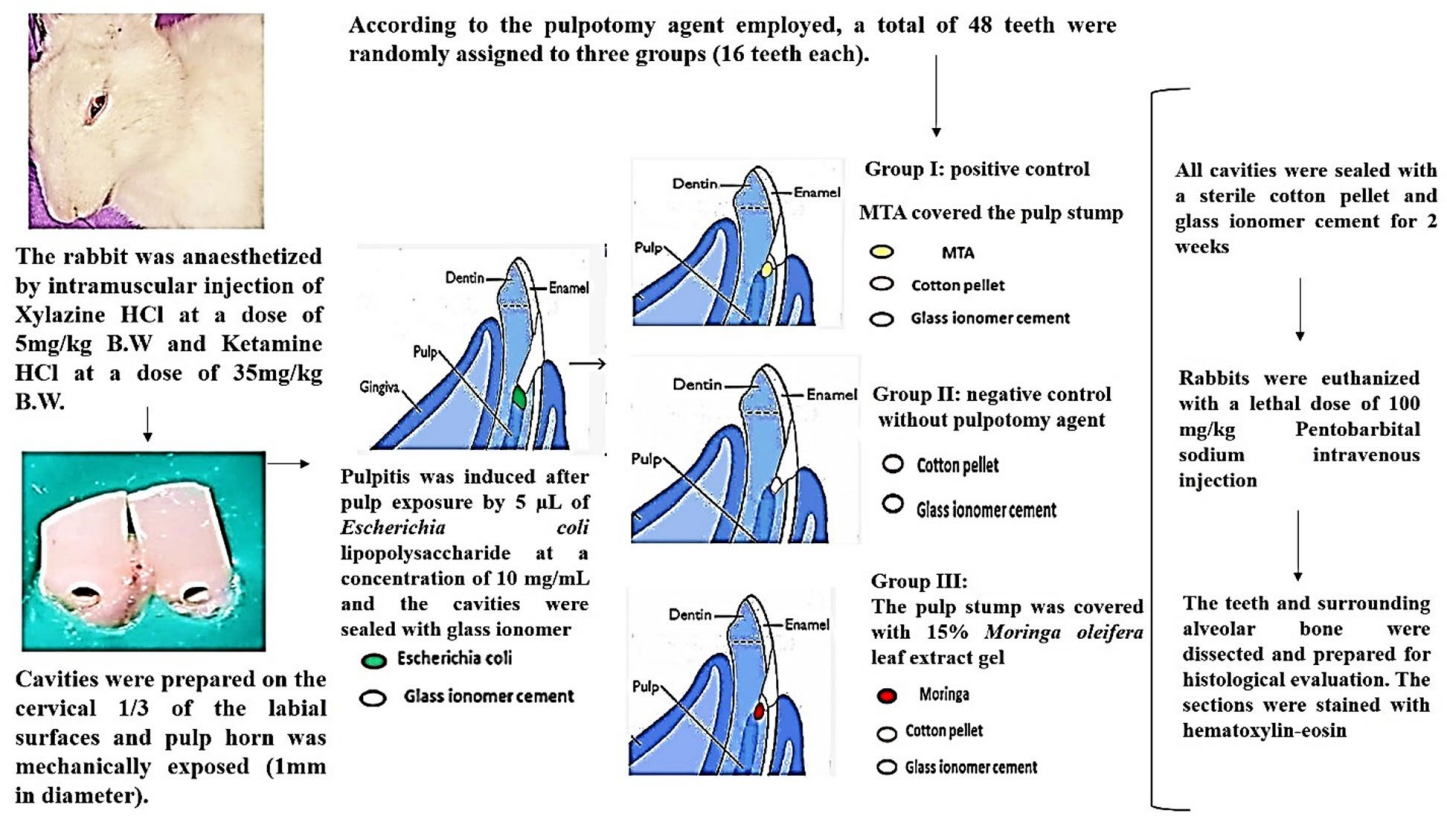
Schematic representation of the methods. Photograph of the rabbit is taken by the authors. The figure was prepared using Microsoft PowerPoint (Microsoft Corporation, Redmond, WA, USA).

The sections were stained with hematoxylin–eosin (H&E) dye and examined by a light microscope Lieca Qwin 500 image analyzer computer system) Lieca, Cambridge, England) at the Pathology Department of the Faculty of Dental Medicine for Girls et al. Azhar University. Photographs with 100 × magnification were obtained. The histological sections were blindly assessed by experienced and independent oral biologist and pathologist. In case of discrepancy between scores, the sections were re-evaluated and a consensus score was reached through discussion. Histological evaluation focused on reparative dentin formation, inflammation and pulp tissue disorganization. An ordinal scoring system was used for the assessment (Table 1), based on a modified version of ISO 10993 and 7405^26^. Schematic representation of the methods is shown in Fig. 1.

**Table 1:** The histological analysis parameters used in the present study.

Reparative dentin formation	Categorization
Score 0	Absence
Score 1	Slight deposition of hard tissue below the exposure site
Score 2	Moderate deposition of hard tissue below exposure site
Score 3	Intense deposition of hard tissue below the exposure site

Cellular inflammatory infiltrate	Categorization
Score 0	Absence of or few inflammatory cells
Score 1	Light inflammatory cells infiltrate as polymorphonuclear leukocytes (PMNLs) and mononuclear leukocytes (MNLs)
Score 2	Moderate inflammatory cells infiltrate
Score 3	Severe inflammatory cells infiltrate or abscess characteristics

Pulp tissue disorganization	Categorization
Score 0	Normal tissue
Score 1	Disorganization of the odontoblast layer only
Score 2	Total disorganization of pulp tissue morphology
Score 3	Pulp necrosis

### Statistical analysis

Statistical analysis was performed using SPSS software program (version 20, SPSS; Inc., Chicago, IL, USA) for Windows. Numerical data were expressed as mean, median, standard deviation, confidence intervals and range. The distribution of the data was evaluated using Kolmogorov–Smirnov and Shapiro–Wilk tests. Due to the non-parametric distribution of data, comparison between the groups was performed using Kruskal Wallis test followed by post hoc test. Qualitative data were presented as count and percentage and were compared between groups using Chi square test. All *P* values are two-sided and a value ≤ 0.05 was considered statistically significant.

## Results

### Group I (MTA group)

**Fig. 2:**
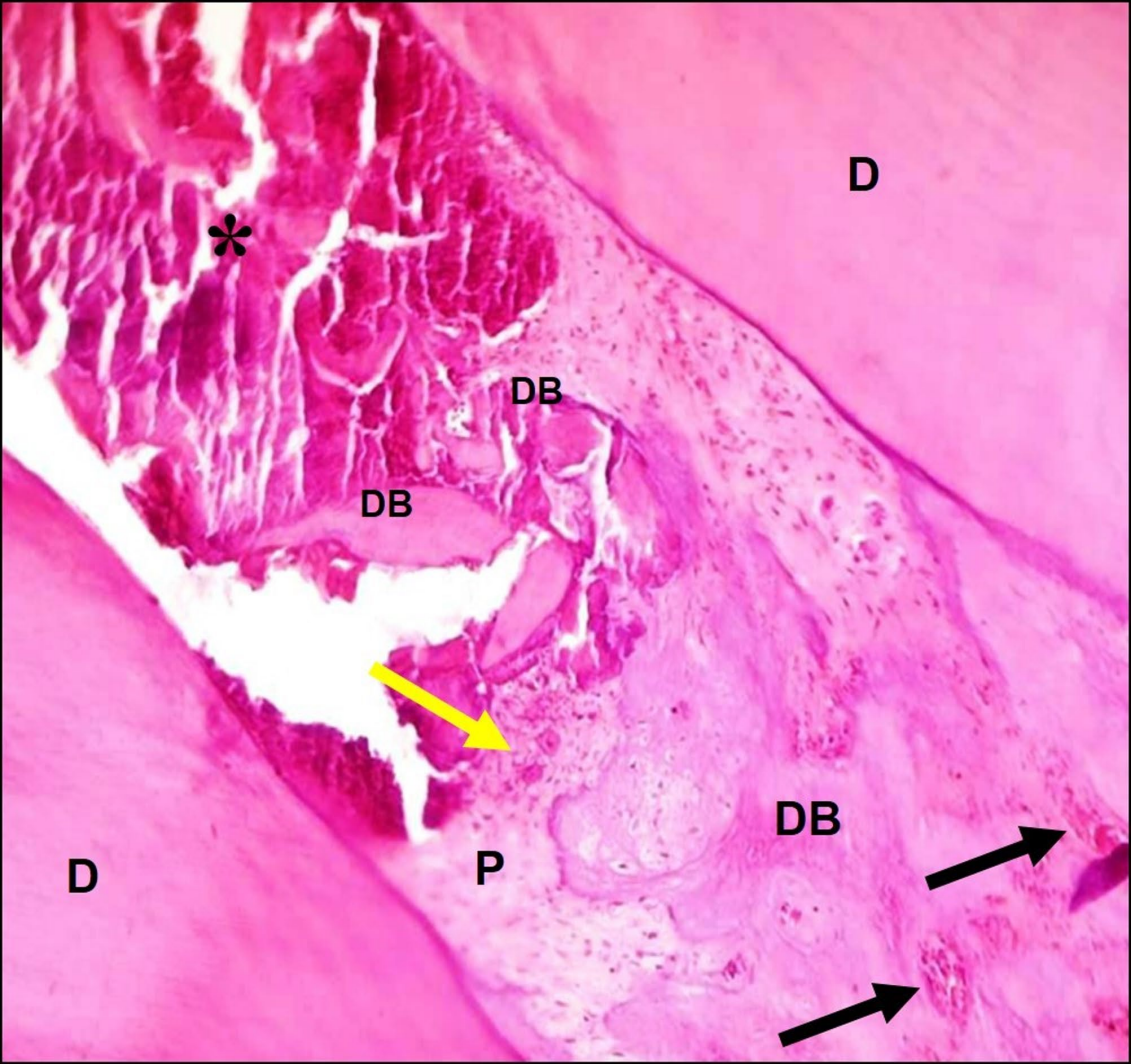
A representative photomicrograph of a rabbit’s incisor in group I (MTA) showing mild inflammatory cells infiltrate (yellow arrow), dilated blood vessels filled with RBCs (black arrows), normal pulp tissue, and intense hard tissue deposition at the amputation site (DB) after 2 weeks follow up (H & E X100). DB: dentin bridge, D: dentin, P: pulp, *asterisk: capping material.

The MTA group showed intense hard tissue deposition at the amputation site with formation of small focuses of hard tissue below this site. The pulp tissue preserved its normal architecture. Mild inflammatory cells infiltrate and dilated blood vessels filled with RBCs were seen (Fig. 2).

### Group II (Negative control group)

**Fig. 3:**
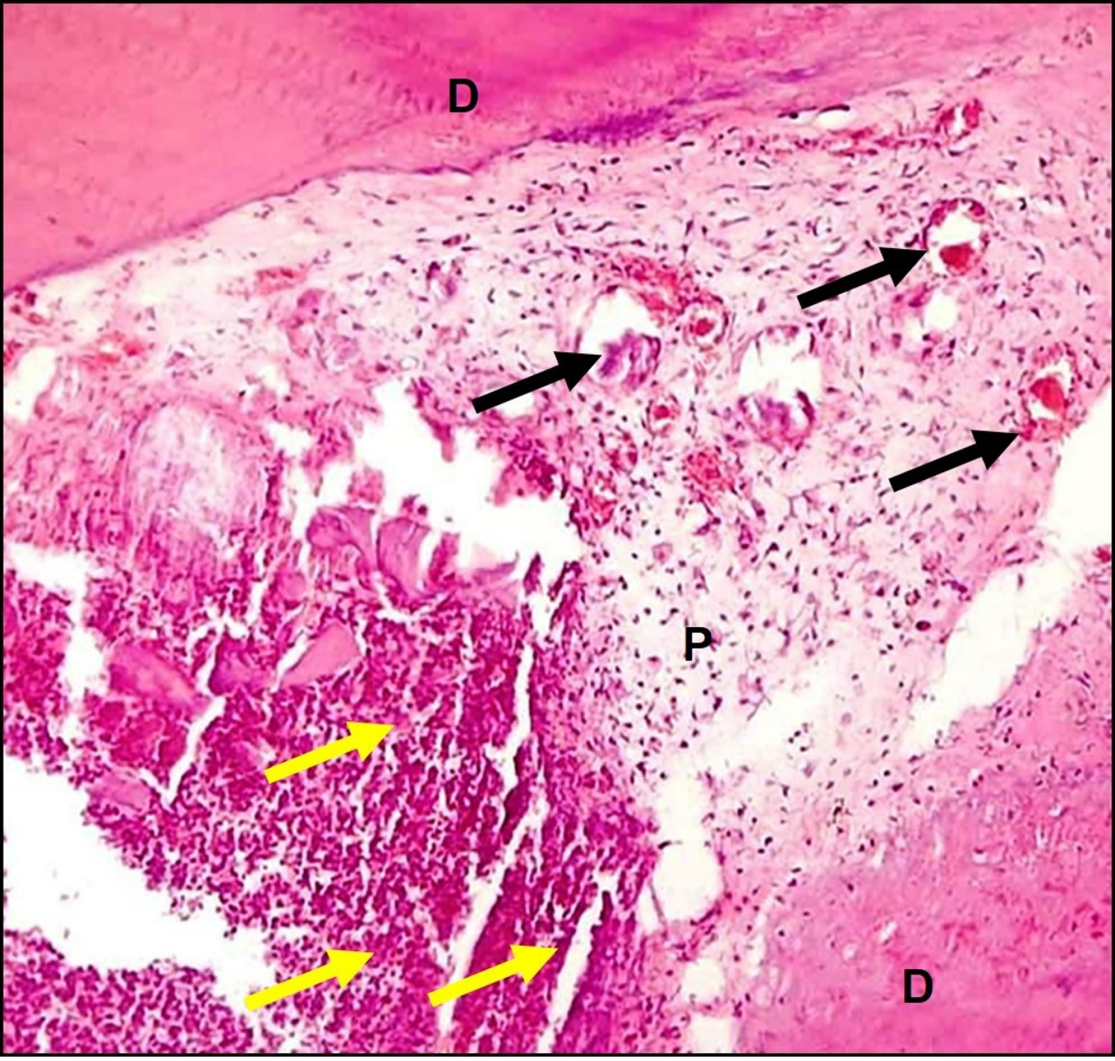
A representative photomicrograph of a rabbit’s incisor in group II (negative control) showing severe inflammatory cells (yellow arrows), dilated blood vessels filled with RBCs (black arrows) and severe pulp tissue disorganization after 2 weeks follow up (H & E X100). D: dentin, P: pulp.

The negative control group exhibited no evidence of hard tissue formation beneath the amputation site. There was severe inflammatory cells invasion accompanied by complete pulp tissue disorganization. The pulp tissue showed dilated blood vessels bloated with RBCs (Fig. 3).

### Group III (15% MO leaf extract gel group)

**Fig. 4:**
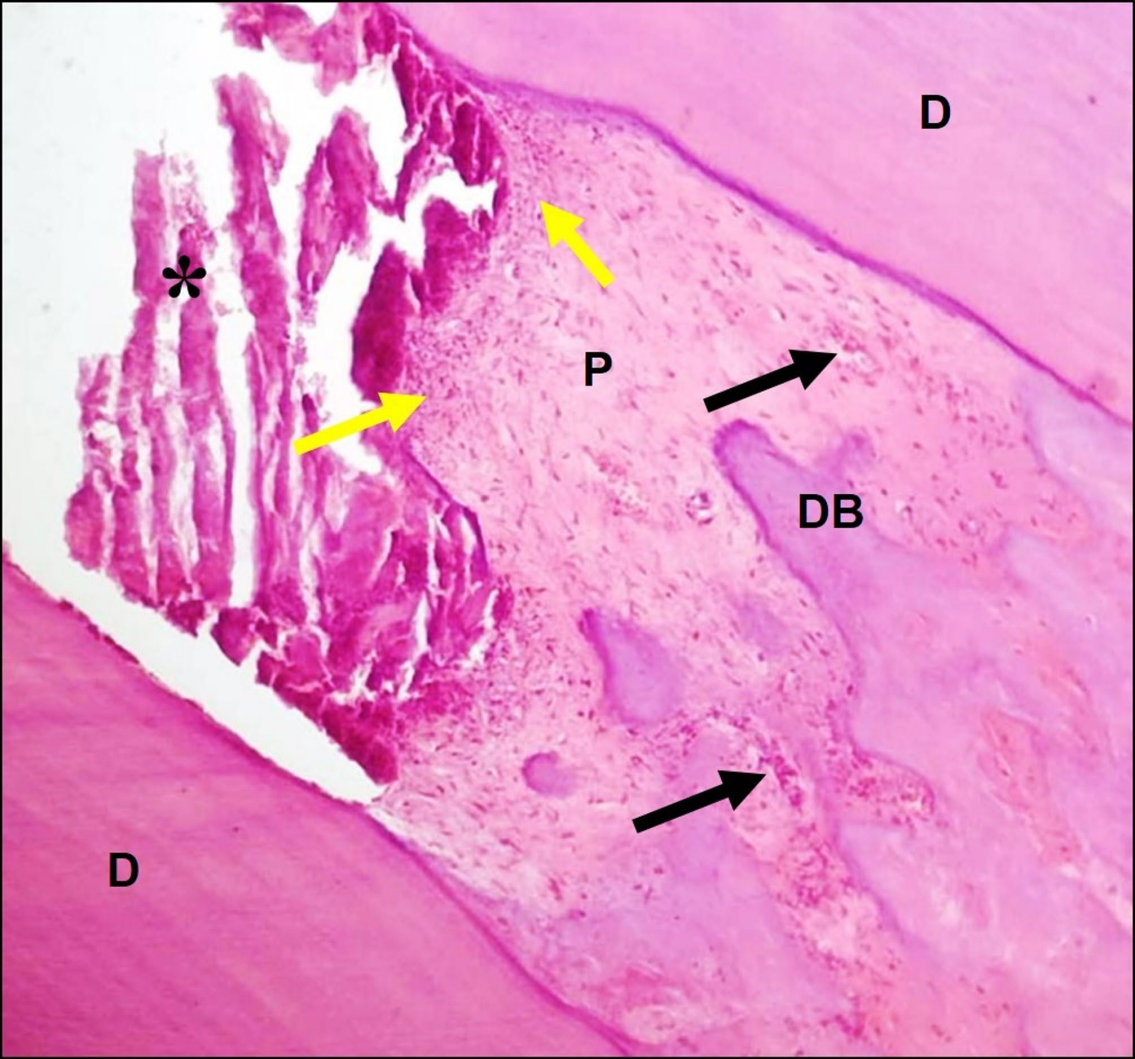
A representative photomicrograph of a rabbit’s incisor in group III (15% *Moringa* leaf extract gel) showing little inflammatory cell infiltrates (yellow arrows), blood vessels filled with RBCs (black arrows), normal pulp tissue, and intense hard tissue deposition at the amputation site (DB) after 2 weeks follow up (H & E X100). DB: dentin bridge, D: dentin, P: pulp, *asterisk: capping material.

The *Moringa oleifera* group demonstrated evident hard tissue formation with preserving the pulp tissue its normal architecture besides having a little inflammatory cell infiltrates mainly in the area adjacent to the amputation site plus presence of blood vessels filled with RBCs (Fig. 4).

### Statistical findings

#### Reparative dentine formation

For groups I and III, no significant difference in reparative dentin formation scores was recorded (*P* > 0.05). Group I exhibited scores 2 and 3 in 50% and 31.3% of the samples, respectively. However, group III showed score 2 and 3 in 50% and 43.8% of the samples. Both groups I and III were significantly different (*P* = 0.000) from Group II. Group II (negative control) exhibited score 0 in all samples (Table 2).

**Table 2:** Descriptive statistics and comparison of frequency of histological parameters scores between groups (chi square test).

Histological parameters	Scores		Groups			Test value	*P* value
			Group I (MTA)	Group II (Negative control)	Group III (15% MO leaf extract gel)		
Reparative dentine formation	Score 0	Count	1	16	0	44.85	0.000*
		%	6.3%	100.0%	0.0%		
	Score 1	Count	2	0	1		
		%	12.5%	0.0%	6.3%		
	Score 2	Count	8	0	8		
		%	50.0%	0.0%	50.0%		
	Score 3	Count	5	0	7		
		%	31.3%	0.0%	43.8%		
Inflammatory cells infiltrate	Score 0	Count	4	1	9	30.9	0.000*
		%	25.0%	6.3%	56.3%		
	Score 1	Count	9	1	5		
		%	56.3%	6.3%	31.3%		
	Score 2	Count	3	7	2		
		%	18.8%	43.8%	12.5%		
	Score 3	Count	0	7	0		
		%	0.0%	43.8%	0.0%		
Pulp tissue disorganization	Score 0	Count	3	0	9	44.33	0.000*
		%	18.8%	0.0%	56.3%		
	Score 1	Count	9	1	7		
		%	56.3%	6.3%	43.8%		
	Score 2	Count	4	3	0		
		%	25.0%	18.8%	0.0%		
	Score 3	Count	0	12	0		
		%	0.0%	75.0%	0.0%		

Significance level *P* ≤ 0.05, *significant.

#### Inflammatory cells infiltrate

As regards inflammatory cells infiltrate, the difference between the three groups was statistically significant (*P* = 0.000). Group III demonstrated the lowest inflammatory cells infiltrate. Group I revealed lower inflammatory cells compared to group II (Table 2) .

#### Pulp tissue disorganization

The difference between the three groups regarding pulp tissue disorganization was statistically significant (*P* = 0.000). High percentage of scores 0 and 1 was recorded in both groups I and III (Table 2), indicating that the pulpal tissue was relatively normal.

## Discussion

Pulpotomy is becoming a more commonly used procedure in treating adult teeth with irreversible pulpitis; it is less aggressive procedure that could improve fracture resistance of the teeth^27^. A previous researches have shown that endodontic therapy causes weakening in the remaining tooth structure^9^. Successful pulpotomy depends on pulp healing capacity and pulpotomy agent biocompatibility^28^. Although MTA has shown excellent properties as a pulpotomy agent, it has difficult handling characteristics, high cost and prolonged setting time^15^. Thus, seeking alternate materials is mandatory. Therefore, this study compared MTA with 15% MO leaf extract gel as pulpotomy agents. The null hypothesis of the study was rejected because 15% MO leaf extract gel as a pulpotomy agent compared to control groups had significant differences in its pulpal responses, regarding new hard tissue formation, inflammation and pulp tissue organization. Effective inflammation control is a critical factor in VPT as it facilitates the differentiation of dental pulp stem cells into odontoblast-like cells and formation of dentin bridge^29^.

Using of MO prevents and treats the periodontal problems, dental caries, and oral mucosal lesions because it reduces pro-inflammatory mediators and prostaglandin production^20,21^. Due to its natural origin and excellent biological actions, we selected MO leaf extract gel in this investigation.

Rabbits were selected as the animal model in the present study due to the anatomical and histological similarities between their pulp tissues and those of human^30^. The New Zealand white rabbits, in particular, was chosen for several reasons; its relatively short life span, the larger size of its teeth compared to other rodents’ teeth, making them more ideal for restorative procedures, and the structural similarity of its teeth and jaw to those of humans^31^. Furthermore, many teeth from the same rabbit can be utilized for any experiment, reducing the overall number of animals required. Teeth within each rabbit were randomly allocated to the experimental group allowing each animal to contribute multiple experimental units and reducing inter animal variability^32^.

Induction of pulpitis in rabbits was performed to mimic the natural progression of inflammation observed in dental caries where bacterial antigens penetrate the pulp through the dentinal tubules. One of these components is LPS, a major component of the outer membrane of Gram-negative bacteria, commonly identified in deep carious lesions and strongly associated with the pathogenesis of pulpitis. It is known that this pathogen-associated molecular patterns stimulates toll-like receptor 4 on dental pulp cells, producing pro-inflammatory cytokines such as interleukin- 1β (IL-1β) and interleukin- 6 (IL-6)^33^.

The follow up period in this study was limited to a short term of 2 weeks. This duration was selected due to the rapid formation of secondary and tertiary dentin in rabbits which is likely attributed to their open-apex and continuous eruption of the teeth throughout life^34^. In rabbits, tooth growth and eruption are balanced by constant dental abrasion from a high fiber diet^35^.

The study’s findings have revealed that in 15% MO leaf extract gel group, 43.8% of the samples showed intense hard tissue deposition and 50% of the samples showed moderate deposition of hard tissue below the amputation site. These findings are in agreement with those of previous studies revealing increased collagen synthesis and mineralization effect of MO leaf extract^24,36^. There is growing evidence that MO extracts possess the ability to stimulate the proliferation of various normal stem cells, support wound healing, facilitate tissue regeneration and enhance angiogenesis. These biological activities, along with antimicrobial properties, suggest that MO extracts may serve as promising therapeutic agents for regenerating the dentin-pulp complex following dental injury. The high content of calcium, phosphate and natural proteins in MO may provide the essential required elements for the mineralization^36^. It has been investigated for its capacity to induce bony growth and support the integration of intraosseous implants. When used in combination with demineralized freeze-dried bovine bone xenograft, MO demonstrated both osteo-conductive and osteo-inductive properties leading to enhanced bone regeneration and socket preservation following tooth extraction. These effects were associated with significantly increased expression of osteocalcin and transforming growth factor-beta 1 (TGF β1)^37^.

Pulpal inflammation interferes with the repair process; inflammation resolution and initiation of the repair process may require a longer duration in pulps affected by bacterial inflammation such as induced by caries, compared to healthy dental pulp^38^. Therefore, the lowest inflammatory cells infiltrate of MO compared to positive and negative controls reflects a favorable pulpal tissue response. MO leaf extract gel contains bioactive compounds as alkaloids, isothiocyanates, flavonoids, saponins, and tannins^24,36^. Therefore, it is considered a remedy for many inflammations at a relatively low cost. Flavonoids help to accelerate the repair process during the inflammatory phase by suppressing the activity of the COX-2 enzyme. High amounts of phenols and tannins that are conjugated with amino acids and gelatin produce an antimicrobial action^39^. The most abundant isothiocyanate has been investigated due to its important antibacterial and anti-inflammatory actions^40^. Our finding is in full agreement with previous studies revealing that 15% MO leaf extract gel can reduce the number of inflammatory cells, shorten bleeding time and increase number of fibroblast and collagen density^24,41^.

The lower anti-inflammatory outcome observed with MTA compared to MO could be related to the biological tissue response associated with its setting reaction. According to previous study, MTA’s alkaline pH and setting reaction may cause a mild inflammatory response at first^42^. The subsequent release of calcium ions aids in mineralization, the formation of calcite crystals, and the stimulation of reparative processes, which are associated with the gradual resolution of inflammation^43^.

Regarding pulp tissue disorganization, both MO and MTA exhibited high percentage of mostly normal pulpal tissues indicating favorable pulpal response, less inflammation and biocompatibility which contribute to preservation of normal pulp architecture. These findings are consistent with a study demonstrating that MO leaf extract exhibited non-cytotoxic effect on dental pulp stem cells and it can induce proliferation and differentiation of the cells suggesting its potential in maintaining organized pulp tissues^20^. In contrast, one study reported that MO had significant cytotoxicity on both human breast adenocarcinoma and normal breast epithelial cell lines. This may be due to different formulation or concentration used in their study^44^.

The novelty of our study lies in the fact that unlike previous investigation on dental pulp studies in rabbits which were conducted under healthy conditions without induced pulpitis^23^, we aimed to simulate a more clinically relevant scenario by inducing inflammation. Such approach will make a foundation for generating scientific evidence supporting the potential clinical use of MO in preserving pulp vitality in cases of irreversible pulpitis. However, our study didn’t succeed in inducing a level of pulpal inflammation severe enough to prevent repair. Future studies should aim to establish a more advanced stage of inflammation for deeper understanding of pulp biology specifically when using different follow up periods (1 week, 2 weeks and 4 weeks). Moreover, further studies should be conducted to explore the mechanism of action of MO on pulp tissue and evaluate its immunohistochemical response.

## Conclusion

Based on the results of the present study, it can be concluded that 15% Moringa Oleifera leaf extract gel demonstrates promising preclinical potential for promoting reparative dentin formation and modulating inflammatory response in pulpotomy. However, the short follow-up period (2 weeks), the use of animal model and the absence of molecular or immunohistochemical analyses represent limitations of the study. Further studies with longer follow-up periods and molecular analyses are required before clinical application can be considered.

## Data Availability

The datasets used and/or analyzed during the present study are available from the corresponding author upon reasonable request.
